# Bilateral kidney matrix stones: a rare case

**DOI:** 10.11604/pamj.2016.25.102.7926

**Published:** 2016-10-20

**Authors:** Mounir Lahyani, Yassine Rhannam, Amine Slaoui, Alae Touzani, Tarik Karmouni, Khalid Elkhader, Abdellatif Koutani, Ahmed Ibn attya Andaloussi

**Affiliations:** 1 Department of Urology B, Ibn Sina Hospital, Rabat, Maroc

**Keywords:** Kidney stone, open surgery, PCNL, matrix

## Abstract

Kedney matrix stones are a rare form of calculi. Flank pain and urinary tract infections (UTI) are the most common presentations of matrix calculi. The diagnosis is usually made at surgery, but some preoperative radiographic findings might be suggestive. Open surgery was the method of choice for treatment. However, combination of ureterorenoscopy and percutaneous nephrolithotomy (PCNL) was found to be safe and effective. We report a rare case of renal and ureteral matrix stones that were diagnosed and treated by open surgery. We also describe its clinical, radiological and therapeutic features through a review of the literature.

## Introduction

Kidney matrix stones (KMS) are a rare form of urinary calculi, presenting a diagnostic and therapeutic dilemma to the practising urologist [1]. By contrast with the normally brittle calcigerous calculi, they are soft, pliable and amorphous [2]. These radiolucent concretions are composed primarily of a noncrystalline mucoprotein matrix.

## Patient and observation

**Figure 1:**
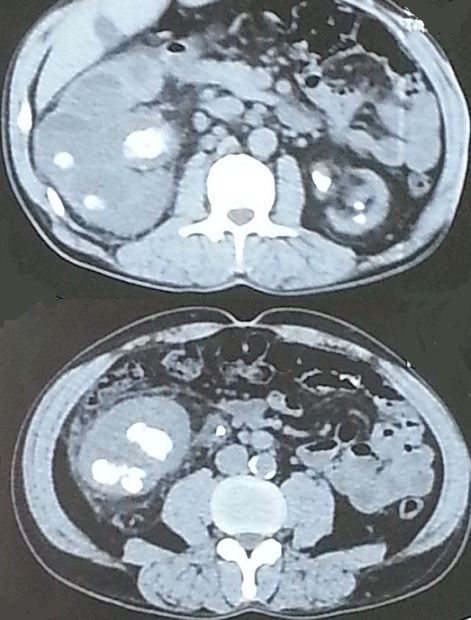
CT showing a right hydronephrosis and hypotrophic left kidney secondary to bilateral renal pelvis and caliceal stones

**Figure 2:**
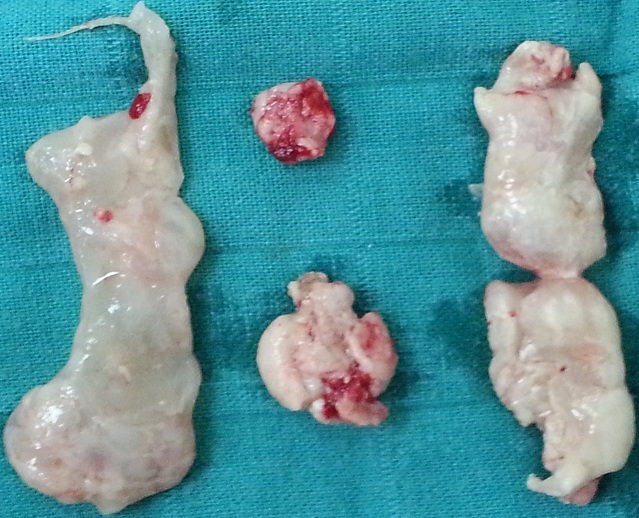
Soft and whitish calculi extracted by renal pelvis lithotomy

Mr A.A. 45 years old, without medical history, complains of right back pain with several episodes of pyelonephritis. Clinical examination was normal. The urinalysis revealed a urinary infection due to Escherichia-coli. The renal function was impaired (Creatinine = 25 mg / l) . X-ray showed no calcium Image of the urinary tract. Ultrasound shows an important right hydronephrosis with multiple slightly echogenic formations in the renal pelvis. CT found an hydronephrosis reducing the renal parenchyma secondary to several large ureteric and caliceal obstructive stones with a low density (520-680 UH) (Figure 1). The left kidney was hypotrophic. The metabolic and phosphocalcic analysis were normal. Antibiotic therapy was started and an open pelvis renal lithotomy was performed. The extracts stones had a soft consistency and look like the ´calamari´ (Figure 2). The patient received two months later a rigid ureteroscopy which allowed the mechanical extraction of residual fragments which has migrated in the lower ureter.

## Discussion

KMS also known as fibrinomas, colloid calculi or albumin calculi, are a rare form of calculi first described in 1908 by Gage and Beal [3]. The matrix is composed of ≈ 64% protein, 9% free sugars, 5% glucosamines, 10% water and 12% inorganic ash . Boyce and Garvey demonstrated that this organic substance is similar, but not identical, to the matrix component of the calcareous stones [4]. According to their analysis, the matrix consisted of mucopolysaccharide (one third) and protein (two thirds); the main components of carbohydrates were hexose and hexosamine, whereas threonine, leucine, serine, tyrosine, arginine and lysine were the most common amino acids of the protein component. By contrast with normal calcigerous renal calculi, which are more common in males, RMS are more common in females [2]. They also have tendency to occur in patients who are stone-formers, especially if they have previously had surgery for stone disease. The main risk factors for this type of stones are previous surgery for stone disease and/or recurrent UTIs, especially due to Proteus mirabilis or Escherichia-coli [5]. Proteinuric patients with glomerulonephritis on dialysis are also at high risk of developing matrix calculi. Seven such patients with considerable persistent proteinuria are reported [5]. The link between KMS and chronic kidney failure is of interest, especially in patients on maintenance hemodialysis, in whom proteinuria and/or UTI or positive urine culture could represent risk factors for developing matrix stones [6]. The clinical presentation of patients with matrix stones is similar to those with calcium nephrolithiasis, flank pain and UTI being the most common symptoms. The imaging of matrix stones can be difficult because no specific radiological investigation is available: in most cases, the diagnosis is only made after surgery [6]. Ultrasound imaging shows a solid structure without the classic hyperechogenicity of stones and acoustic shadowing, depending on the amount of mineralization.

The computed tomography (CT) appearance of KMS varies, depending on mineral volume, composition and internal distribution. Some authors described egg-shaped matrix stones with a mineral rim and soft tissue center [1]. MRI shows a hypointense signal in T1-weighted images and a slight hyper-intense signal in T2-weighted images. No obvious contrast enhancement was found after gadolinium administration in T1-weighted images [7]. Histological examination of these calculi shows laminar concentric rings of organized matrix with an orderly, layered deposition of minerals. In the past, open surgery was the preferred technique [2]. Due to the soft consistency of the stones, methods like milking of the proteinaceous material from the ureter into the bladder or use of a special bottle brush to clear the pelvicalyceal system were used during this procedure [8]. Nowadays, the most appropriate choice in the treatment of KMS is the endourological option. Both procedures, the antegrade (PCNL) and the retrograde (RIRS) one, have been used by various authors [9]. PCNL may be safe and effective to remove matrix calculi in a single session, while the ureteroscopic approach is often inadequate with large bulk of stones. SWL is usually ineffective due to the stones gelatinous component and a lack of breakable mineral content [1]. Fortunately, these stones have a very low recurrence rate once the stone is completely cleared. Prophylaxis with antibiotics is believed to be effective to avoid KMS recurrences. Several preparations for lowering urine pH might also be useful in treating patients with infected renal stones [10]. In future, prospective multicentre studies are necessary to provide insights into the aetiopathogenesis of this rare entity. Histochemical investigation can also provide an insight into the possible sequence of events in normal calculogenesis [2].

## Conclusion

KMS are a rare entity. The diagnosis is usually made at surgery, but some preoperative radiographic findings might be suggestive. Open surgery was the method of choice for treating these patients in the past. However, recently it was replaced by endourological intervention. Washing the pelvis and prophylaxis with antibiotics are believed to be effective to avoid recurrences.
